# The Effect of Digital Health Interventions on Parents’ Mental Health Literacy and Help Seeking for Their Child’s Mental Health Problem: Systematic Review

**DOI:** 10.2196/28771

**Published:** 2022-02-10

**Authors:** Daniel Peyton, Marquelle Goods, Harriet Hiscock

**Affiliations:** 1 Murdoch Children's Research Institute Parkville Australia; 2 Department of Paediatrics, University of Melbourne Melbourne Australia; 3 Health Services Research Unit, The Royal Children's Hospital Melbourne Australia

**Keywords:** child, mental health, systematic review, caregiver, health literacy, digital health

## Abstract

**Background:**

Many children with mental health problems do not receive professional help. Despite the frequent use of digital health interventions (DHIs) such as websites or web-based service navigation platforms, their effects on parents’ mental health literacy, help seeking, or uptake of professional services are unclear.

**Objective:**

This study aims to provide a systematic review and narrative synthesis to describe whether DHIs improve the aforementioned parental outcomes.

**Methods:**

Databases, including CINAHL, Embase, MEDLINE OVID, PsycINFO, and PubMed (2000-2020), were accessed. Studies were included if they evaluated quantitative changes in mental health literacy, help seeking, or the uptake of services by parents of children with mental health problems. Theoretical frameworks, sample sizes, participant demographics, recruitment, interventions, DHI use, results, and health economic measures were used for data extraction.

**Results:**

Of the 11,379 search results, 5 (0.04%) studies met the inclusion criteria. One randomized controlled trial found the reduced uptake of services after using a DHI coupled with a telephone coach for a child’s behavioral problem. Of 3 studies, 2 (66.7%) found statistically significant improvement in mental health literacy for attention-deficit/hyperactivity disorder but had no control group. One study found nonsignificant improvement in mental health literacy and help-seeking attitudes toward anxiety and depression compared with those in active controls. All studies were rated as having a high or serious risk of bias. Search results were affected because of a single reviewer screening articles, overall low-quality studies, and a lack of consistent nomenclature.

**Conclusions:**

There is no high-quality evidence that DHIs can improve parents’ mental health literacy, help seeking, or uptake of services. More research is needed to evaluate DHIs by using rigorous study designs and consistent measures.

**Trial Registration:**

PROSPERO International Prospective Register of Systematic Reviews CRD42020130074; https://www.crd.york.ac.uk/prospero/display_record.php?ID=CRD42020130074

## Introduction

### Background

Mental health problems are common among children [1,2]. They include internalizing problems, such as anxiety and lowered mood, and externalizing problems, such as hyperactivity, oppositional defiance, and aggression. Around half of these problems can progress to mental health disorders that are associated with adverse outcomes, including early school dropout, criminal justice system involvement, lower life satisfaction, poorer relationships, and lower earning potential [3-9]. Fortunately, there is a range of evidence-based treatments that have been shown to improve mental health problems in children, including the use of websites or web-based programs or other digital health interventions (DHIs) [10-13]. A DHI can be defined as the digital delivery of health information, such as through websites or apps, for health-related purposes [14]. Many of these treatments, including those delivered by DHIs and face-to-face interventions, focus on improving parenting—a key modifiable risk factor for these problems [15]. Despite treatments being available, many children with mental health problems do not receive professional help [2,16-18].

**Figure 1:**
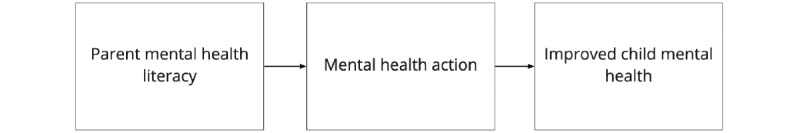
Link between parent mental health literacy and child mental health outcomes (adapted from a study by Jorm [22]).

There are several recurrent barriers that prevent children receiving professional help. These barriers can be viewed along the help-seeking process, as parents need to recognize their child’s problem and acknowledge their need for additional support, be aware of treatment options, overcome stigma in accessing treatment, and ultimately access available services or treatments [2,19,20]. A lack of problem recognition and awareness of available treatments reflect inadequate mental health literacy, which has been defined as the “knowledge and beliefs about mental disorders which aid their recognition, management or prevention” [21]. Mental health literacy is important because it is linked to actions and mental health outcomes [22]. For children, especially young children, parents play a large role in recognizing the child’s problem and facilitating help seeking (Figure 1).

Ideally, we should be able to improve parents’ knowledge of mental health problems in children and where to find available and accessible services to help their children. This could be done by improving their mental health literacy, a known modifiable factor of help seeking [23]. However, previous research on interventions designed to improve mental health literacy and help seeking has been hampered by a lack of consistent measures of mental health literacy and a lack of focus on parents [22,24,25]. For parents, a US study with 165 children with mood disorders and other mental health comorbidities showed that face-to-face mental health literacy interventions can improve the quality of services accessed by families compared with waitlist control. The quality of services was measured by consensus among a group of blinded expert clinician researchers [26]. However, this intervention was intensive (8 group sessions lasting 90 minutes each) and may have been affected by attrition bias, as only 74% of participants completed the 18-month follow-up. In addition, several families dropped out of the waitlist control group after their child’s symptoms improved, underscoring the need for controlled trials to account for the natural history of some mental health problems improving over time.

Digital delivery of this educational material to parents, such as through a DHI, may prove to be an effective, accessible, scalable, and desirable way to improve parents’ mental health literacy and help seeking. Most parents search the web for health information and seek out the lived experience of other parents through forums, such as those on Facebook [27,28]. As parents seek out this information on the web, money and resources are devoted to building websites, apps, and platforms to help parents better understand their child’s mental health and where to receive help. Child mental health websites, such as childmind.org, can have enormous reach with a recent mental health campaign reaching 275 million people [29].

The World Health Organization states that DHIs have many perceived benefits, including enhanced reach, accessibility, scalability, desirability, reduced stigma, and perceived cost-effectiveness [14]. DHIs’ perception of cost-effectiveness comes from the potential for near-infinite scalability at low cost and targeted early intervention [14,30,31]. However, data on cost-effectiveness are rarely collected, despite recommendations to measure the economic impact as part of any DHI evaluation [32,33].

DHIs have been shown to improve mental health literacy in adults, based on the findings of 2 systematic reviews [34,35]. However, these reviews, which included a combined total of 28 studies, only included 1 study with parents.

The single-parent study was a randomized controlled trial that found that a convenience sample of parents recruited from a single workplace improved their mental health literacy from a DHI [36]. This lack of focus on parents in previous reviews is important because parents are the agents of change for their child’s mental health. Unlike adults seeking help for themselves, parents’ willingness to receive help for their child’s mental health problem is influenced by unique factors, such as whether the child participates in mental health treatment, or whether the treatment is framed in terms of child development [37,38]. With half of all adult mental health disorders originating in childhood, it is crucial to determine how DHIs can improve parents’ mental health literacy, help seeking, and uptake of mental health services for their children [9].

However, there have been no consistent positive effects on parental help-seeking attitudes, with some low-quality studies finding a positive effect of DHIs, but most found no effects [34,35]. Studies in these 2 reviews had some limitations, specifically the common use of convenience sampling, the predominant focus on young people, lack of consistent measures, and low-quality evidence.

Recently, a universal education program delivered via SMS text messaging improved mental health literacy in the parents of adolescents compared with care as usual control. However, this study did not include parents of younger children or parents who were identified as having an adolescent with a mental health problem, who may be more likely to benefit from an intervention that facilitates help seeking [39].

Little is known about the effects of a DHI on the mental health literacy of parents, especially parents of young children, and even less is known about the effects on help seeking and uptake of services and cost-effectiveness. This is despite the frequent use of DHIs by parents and low uptake of services among many children with a mental health problem.

### Objectives

In this study, we aim to conduct a systematic review of the literature to understand (1) whether DHIs targeting parents of children aged 2 to 12 years with a mental health problem improve mental health literacy and (2) whether the use of DHIs is associated with changes in parental help seeking or uptake of mental health services for their child. We also aim to report the cost-effectiveness of such DHIs.

## Methods

The systematic review was registered with PROSPERO (CRD42020130074). We conducted and reported a systematic review according to the PRISMA (Preferred Reporting Items for Systematic Reviews and Meta-Analyses) guidelines [40].

### Eligibility

We included studies that evaluated a DHI delivered directly to parents of children aged 2 to 12 years, with quantitative data reporting on outcomes of mental health literacy (specifically knowledge of treatment), help seeking (attitudes, intentions, and behaviors), or uptake of mental health services. Quantitative data were chosen to narratively synthesize the impact of DHIs on mental health literacy, help seeking and uptake of services.

For this review, we defined a DHI as a consumer-facing intervention using information communication technology targeting parents. The intervention could deliver information as a static webpage, a web-based parenting program, a web-based social network, a native mobile app, or other content delivered using digital means (other than telehealth). This definition was included in the PROSPERO registration.

We included DHIs targeting children with and without a mental health condition as long as the DHI was delivered as part of a program where some families were identified as having a mental health concern for their child. We included children aged 2 to 12 years. This age range was selected because of their likely dependence on parents to receive help for their mental health and the long-term impact of these early years on the well-being into adulthood [9]. We required a minimum of 1 outcome question on mental health literacy focusing on any of the following: knowledge of treatment, help seeking, or uptake of services.

Study designs included randomized controlled trials, quasi-randomized trials, and uncontrolled single-cohort studies. We restricted our analysis to articles published between January 2000 and December 2020 and written in English. We excluded conference proceedings and gray literature.

### Data Sources and Search Strategy

We developed our search strategy after consultation with a research librarian at The Royal Children’s Hospital, Melbourne, Australia. A pilot search was performed in MEDLINE OVID, followed by a review of keywords and further development of the search strategy. We searched the electronic databases CINAHL, Embase, MEDLINE OVID, PsycINFO, and PubMed in late 2019 and repeated the search in January 2021 to identify any more recent publications.

We also reviewed the reference list of the included studies to identify additional studies for full-text review. All search results were compiled in Endnote and then exported to Covidence for screening. The search strategy used for all the databases is given in Multimedia Appendix 1.

### Study Selection

One author (DP) screened the titles and abstracts of all articles produced from the search against the eligibility criteria. The full text of the remaining articles was obtained and screened again against the inclusion criteria. Any concerns about study eligibility were resolved in discussions with the supervising author (HH) during fortnightly supervision meetings. If there was insufficient evidence from the full-text study on whether it met the inclusion or exclusion criteria, DP attempted to contact the authors to obtain relevant information.

### Data Collection Process

Two authors (DP and MG) independently extracted data from the included studies using a pre-existing data collection form for intervention reviews from Cochrane [41].

### Data Items

Data extracted included study design; number of participants; type of comparison (where relevant); setting; recruitment; age and sex of participants and their children; the intervention, including the theoretical basis (a factor that may influence the success of a help-seeking intervention) [42] and measures of DHI use; outcome measures and whether they are validated measures; results; and economic outcomes. The data extracted were compared for accuracy, and the supervising author (HH) resolved any disagreements. Where possible, we calculated the effect sizes of the interventions and included these in Table 1.

**Table 1:** Primary outcomes of the interventions.

Study	Design	Sample, n	Intervention	Timing of measures	Primary outcome	Measure	Outcome	*P* value	Validated measure
Montoya et al [43]	Pre or post single cohort	35	DISCERN tool assessing popular Spanish websites about ADHD^a^ treatment	Unspecified time points pre, post parents using the DISCERN tool	Mental health literacy: ADHD specific knowledge and motivation for treatment	The ADHD-knowledge and motivation for treatment questionnaire (ADHD-KMT). Basic knowledge subscale	Pre: mean 49.09 (SD 9.46) Post: mean 63.21 (SD 9.45) Cohen *d*=1.49	<.01	No
Ossebaard et al [44]	Pre or post single cohort	195	Web-based decision aid on ADHD treatment	Pre, post intervention, though exact timing unclear	Mental health literacy: ADHD knowledge and treatment	“Would you please rate your knowledge on ADHD and its treatment possibilities” with a response on a 1-10 numerical scale	Pre: mean 6.2 (SD 1.9) Post: mean 6.5 (SD 1.9) Cohen *d*=0.16	.60	Unclear
Ryan et al [45]	Pre or post single cohort	172	Information-based website on ADHD management	Baseline: 28 days postbaseline	Mental health literacy: ADHD knowledge	ADHD Knowledge and Opinions Survey-Revised (AKOS-R) – adapted Lower score (min: 30; max: 60)=higher knowledge	Wilcoxon signed rank test showed a statistically significant moderate increase in knowledge; Z=−4.799; Cohen *d*=−0.503	<.01	No
Sapru et al [46]	Nonrandomized controlled trials	27	3× PowerPoint presentations emailed to participants	Pre and post intervention, though exact timing unclear	Mental health literacy and help-seeking attitudes for depression	Understanding mood disorders questionnaire Lower incorrect score=higher knowledge	Median number of incorrect scores: Intervention: Pre 7, post 1; Control: Pre 7.5, post 4 Within-group difference (pre or post) in PowerPoint group: Wilcoxon signed-rank test showed statistically significant improvement in responses (Z=−2.30; *P*=.04) Comparison between PowerPoint group and control (in-person group): One-way ANOVA showed no statistically significant improvement difference in responses	Within-group difference (pre or post) in PowerPoint group: *P*=.04 Comparison between PowerPoint group and control (in-person group): *P* value not reported	Not reported
				Pre, post intervention, though exact timing unclear	Mental health literacy and help-seeking attitudes for anxiety	Understanding of anxiety disorders questionnaire Lower incorrect score=higher knowledge	Median number of incorrect scores: Intervention: Pre 9, post 2; Control: Pre 6.5, post 3.5 Within-group difference (pre or post) in PowerPoint group: Wilcoxon signed-rank test showed statistically significant improvement in responses (Z=−2.30, *P*=.04) Comparison between PowerPoint group and control (in-person group): one-way ANOVA showed no statistically significant improvement difference in responses	Within-group difference (pre or post) in PowerPoint group: *P*=.04 Comparison between Power Point group and control (in-person group): *P* value not reported	Not reported
Sourander et al [47]	Randomized controlled trial	464	Strongest Families’ Smart website and 11× weekly 45-minute telephone coaching sessions	6 months, 12 months, and 2 years after randomization	Uptake of services in the past 6 months	Past service use evaluated using a yes or no question: “asking the parents if the child had received any behavioural treatment in the last 6 months”	Number of participants reporting uptake of services: Intervention: 28 (18%); Control: 46 (28%); OR 1.8 [95% CI 1.1-3.1]	.02	No

^a^ADHD: attention-deficit/hyperactivity disorder.

### Risk of Bias

The included studies were assessed for quality against 1 of 2 instruments. For nonrandomized studies, we assessed the risk of bias using the Risk of Bias in Nonrandomized Studies of Interventions tool [48]. For randomized studies, we assessed bias using the revised Cochrane tool for assessing risk of bias in randomized trials [49]. The quality assessment was conducted independently by DP and MG. They compared their assessments and resolved any disputes by discussion or through the input of the supervising author (HH).

### Summary Measures

Whenever possible, we presented the outcome data of mental health literacy, help seeking, and uptake of services consistently, with parametric continuous data compared using means, nonparametric continuous data presented using medians, and categorical data presented as proportions. We also attempted to group the outcome data by validated and unvalidated measures.

### Synthesis

Owing to the heterogeneity in outcome measures, we could not conduct a meta-analysis. Accordingly, we used a narrative synthesis to describe the effects of the DHIs.

## Results

### Search Results

**Figure 2:**
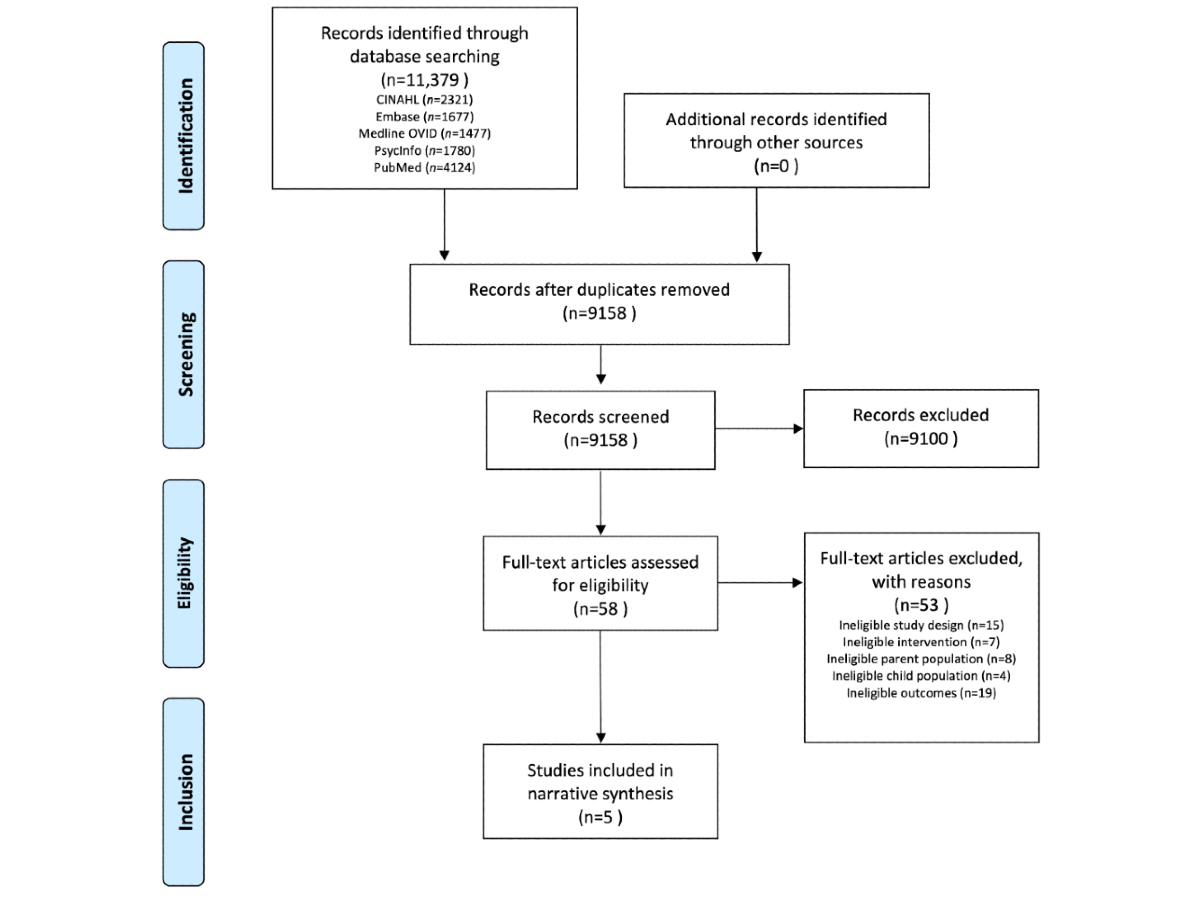
PRISMA (Preferred Reporting Items for Systematic Reviews and Meta-Analyses) flow diagram of search results and study selection.

Through the search strategy detailed in the previous section, a total of 11,379 potentially eligible articles were identified. Of the 11,379 articles, 5 (0.04%) met all inclusion and exclusion criteria (Figure 2). The primary author (DP) reviewed the reference list of these included studies, which revealed no additional studies meeting inclusion and exclusion criteria.

### Description of Included Studies

Of the 5 included studies, 1 (20%) was a randomized controlled trial [47], 1 (20%) was a nonrandomized trial of 2 interventions [46], and 3 (60%) were uncontrolled before and after studies [43-45] (Table 2).

All 5 studies were published between 2010 and 2018. There were 893 participants across the 5 studies, with the number of participants ranged from 27 to 464. The mean age of the children ranged from 4 to 10 years across the 5 studies. All studies were published in Europe or North America. Outcome measures included knowledge of attention-deficit/hyperactivity disorder (ADHD) treatment, knowledge and help seeking for anxiety and depression, and uptake of treatment for a child’s behavioral problem.

A total of 3 studies included participants with concerns about, or a recent diagnosis of, ADHD [43-45]. Another study included only participants who were parents of children with high-level disruptive behavior on the Strengths and Difficulties Questionnaire and who recognized that their child had a problem [47]. The final study included participants who were referred to a tertiary center for management of the child’s anxiety or depressive disorder, although the authors did not describe how the disorder had been diagnosed [46].

Participants were sampled using a variety of techniques. Of the 5 studies, 2 (40%) used consecutive sampling techniques to approach participants attending a scheduled universal health appointment [47] or a tertiary hospital mental health outpatient clinic [46]; 1 (20%) used a convenience sample of participants who had already attempted to access the intervention evaluated in the study [44]; and 1 (20%) used a convenience sample in which participants were selected by their child’s physician or from a local advocacy group [43].

**Table 2:** Study description.

Study	Country	Design	Sample, n	Participants	Recruitment	Intervention	Comparator	Theoretical basis for the intervention	Digital health intervention use	Economic outcomes
Montoya et al [43]	Spain	Single cohort pre or post study	35	Parents of children with a recent diagnosis of ADHD^a^	Parents selected by their child’s physician or from a local advocacy group	Use of the DISCERN tool to assess the quality of 10 popular Spanish websites about ADHD treatment	Nil	Not reported	Not reported	Not reported
Ossebaard et al [44]	Netherlands	Single cohort pre or post study	195	Parents of children with a recent diagnosis of ADHD	The web-based decision aid invited visitors to the website to participate in the study	Web-based decision aid on ADHD treatment	Nil	Yes	About 7500 unique visits About 6 minutes on site About 8-9 clicks to navigate	Not reported
Ryan et al [45]	United Kingdom	Single cohort pre or post study	172	Parent or carer of a child with confirmed or suspected ADHD	Invited to attend if attending one of 3 pediatric outpatient clinics for suspected or confirmed ADHD	Information based website on ADHD management	Nil	Not reported	Never used the website: 62 (41%) 1-2 times: 50 (33%) 4-5 times: 27 (18%) 5-6 times: 6 (4%) 7+ times: 8 (5%)	Not reported
Sapru et al [46]	Canada	Prospective nonrandomized controlled trial before and after study	27	Families referred to a tertiary hospital for management of a mood or anxiety disorder	Families on a waitlist for outpatient treatment of depression or anxiety were invited to attend	3× PowerPoint presentations emailed to participants	3 × 1-hour in-person group family psychoeducation sessions	Yes	PowerPoint presentations completed: mean 2.7 (SD 2.7) Control group: mean 3.75 (SD 2.3)	Not reported
Sourander et al [47]	Finland	Prospective randomized controlled trial	464	Parents of children with high level disruptive behavior at a universal 4-year-old health check	Families attending a universal 4-year-old health check were screened and invited to attend	Strongest families smart website and 11× weekly 45-minute telephone coaching sessions	Brief website on positive parenting strategies and single 45-minute telephone coaching session and standard care	Not reported	Not reported	Not reported

^a^ADHD: attention-deficit/hyperactivity disorder.

### Description of the Included Interventions

Of the 5 interventions, 4 (80%) were delivered on the web through a website [43-45,47] and 1 (20%) was delivered via a series of PowerPoint presentations [46]. These PowerPoint presentations were emailed to each family every week for 3 weeks. The topics of the three PowerPoint presentations were (1) introduction and treatment options, (2) interpersonal illness and communication skills, and (3) problem solving and personal reflection [46].

A total of 2 (40%) web-based interventions were delivered with a cointervention [43,47]. One (20%) of these cointerventions consisted of 11 consecutive weekly telephone coaching sessions, in addition to access to the Strongest Families Smart Website [47]. This website features 11 sessions containing tailored content, exercises, and instructional videos and requires parents to complete knowledge and experience-based questions. This content is designed to help parents develop skills to promote positive behavior and a positive relationship with their children [47]. Another study by Montoya et al [43] used a cointervention. In this study, parents evaluated popular ADHD websites against the DISCERN instrument [50] to assess the quality of written consumer health information available on ADHD treatment [43].

The remaining 2 (40%) interventions consisted of a website focused on ADHD [44,45]. A study by Ossebaard et al [44] trialed a web-based decision aid designed to help support parents and caregivers through the decision-making process of ADHD treatment. The average visitor, which included participants and nonparticipants, visited the website for an average of 6 minutes [44]. The final ADHD website contained information on the management of ADHD [45]. The website was funded by the pharmaceutical company Shire, which was disclosed to the participants. The participants could access the website for 1 month, and most of the participants accessed the website once or twice during that time [45]. For these 2 ADHD websites, postintervention outcomes were measured immediately following the intervention [44], 30 days after the intervention started [45], or 2 years after the intervention commenced [47].

Of the 5 studies, 2 (40%) did not specify precisely when they recorded postintervention outcomes [43,46].

### Effect on Mental Health Literacy, Help Seeking, and Uptake of Services

Mental health literacy outcomes were the most common outcome assessed by the included studies, with 80% (4/5) of the studies measuring some form of mental health treatment knowledge (Table 1). The most common mental health problem assessed by the knowledge measures was ADHD [43-45], followed by depression and anxiety knowledge and help-seeking attitudes studied by Sapru et al [46]. Only 1 (20%) study measured the parent-reported uptake of mental health services [47].

### ADHD Knowledge

Despite 60% (3/5) of the studies intending to measure ADHD knowledge and all through survey responses, each study used a different measure. None of these measures were validated. An adapted version of a validated measure was used by Ryan et al [45], but the authors did not provide a description of how it had been adapted and whether it was still valid. All of the ADHD knowledge studies were uncontrolled pre-post studies, and all showed an improvement in parent ADHD knowledge scores, 2 (40%) of which were statistically significant [42,49].

In addition, changes in knowledge among those who accessed the website and those who did not were assessed by Ryan et al [45]. Their study [45] showed that those who accessed the website at least once had a moderately significant improvement in knowledge compared with those who never accessed the *ADHD and You* website.

Of note, evaluation of a web-based decision aid by Ossebaard et al [44] was affected by a large number of missing data. From the 7500 unique views to the site, all of whom were invited to participate in the study, only 195 participants were enrolled, leading to potential selection bias. In addition, of these 195 participants, only 12 (6.2%) provided outcome data before and after the intervention, leading to potential attrition bias.

### Depression and Anxiety Knowledge and Attitudes to Help Seeking

The only study that evaluated anxiety and depression-based mental health literacy and help-seeking attitudes was carried out by Sapru et al [46]. One measure was used for anxiety, and another for depression, with each measure assessing both knowledge and help-seeking attitudes within the same instrument. It was not reported whether these tools had been validated for this population.

Both the anxiety and depression measures showed an improvement in median scores of the intervention (web-based) compared with those of the control (in-person) group, although this difference was not significant in a small sample size.

Missing data and high attrition rates were again common, with outcome data provided for only 38% (5/13) of the intervention participants and 57% (8/14) of the control participants. The authors did not report why so many families failed to initiate or complete the programs and outcome measures. Two of the authors were contacted but did not provide further clarification on reasons for the missing data.

### Uptake of Mental Health Services

Only 1 (20%) study measured the uptake of mental health services, which was also the largest study and had the longest follow-up of 2 years [47]. A study by Sourander et al [47] asked parents to self-report whether they had received any behavior treatment for their child in the previous 6 months. This measure was recorded at 6, 12, and 24 months after starting the 11-week intervention. The authors did not report whether this measure had been validated. Fewer parents in the intervention group, consisting of a website and 11 weekly telephone coaching sessions, reported that their child had accessed behavioral treatments (28/160, 17.5% participants) than did parents in the control group (46/164, 28% participants; odds ratio 1.8, 95% CI 1.1-3.1; *P*=.02). This reduction in the uptake of behavioral treatments occurred in the context of a small but significant improvement in the child’s behavior in the intervention group compared with the control group.

### Cost-effectiveness

No studies reported on the cost-effectiveness or costs of the DHIs.

### Assessment of Risk of Bias

One randomized controlled trial was rated as having a high risk of bias in 1 domain because of missing data, giving it an overall rating of high risk (Table 3) [47].

A total of 4 study designs were nonrandomized, with 3 (75%) of these studies [43,45,46] rated at serious risk of bias and 1 (25%) [44] rated at critical risk of bias (Table 4). The studies were rated at serious risk of bias because of a lack of identification of, or control for, potential confounders; potential for bias in selection of participants; and lack of objective outcome measures. The large number of missing participants also contributed to attrition bias and subsequent critical risk ratings.

**Table 3:** Risk of bias of randomized studies using the Cochrane tool for assessing risk of bias in randomized trials (RoB 2).

Study	Randomization process or selection bias	Deviations from intervention	Missing outcome or attrition bias	Measurement of outcome or detection bias	Selection of reported result or reporting bias	Overall
Sourander et al [47]	Low	Low	High	Some concerns	Some concerns	High

**Table 4:** Risk of bias in nonrandomized studies using the Risk of Bias in Non-randomized Studies–of Interventions (ROBINS-I) tool.

Study	Confounding	Selection of participants	Classification of interventions	Deviations from intended interventions	Missing data	Measurement of outcomes	Selection of reported result	Overall
Montoya et al [43]	Serious	Low	Low	Low	Low	Moderate	Moderate	Serious
Ossebaard et al [44]	Serious	Critical	Low	Low	Critical	Serious	Moderate	Critical
Ryan et al [45]	Serious	Low	Low	Low	Moderate	Serious	Moderate	Serious
Sapru et al [46]	Serious	Serious	Low	Low	Moderate	Moderate	Moderate	Serious

## Discussion

### Principal Findings

This study identified 5 studies of DHIs for parents of children with a mental health problem, measuring changes in mental health literacy, help seeking, or uptake of services.

Of those measuring mental health literacy, 80% (4/5) of the studies showed an improvement in parent knowledge. However, most of these studies focused on ADHD knowledge and were of low quality.

Of the 5 studies, 1 (20%), using a very small sample size of parents, measured both mental health literacy and help-seeking attitudes and used a nonrandomized control group, showing a nonsignificant trend to improved knowledge and help-seeking attitudes for child’s anxiety and depression. For this study, the mental health literacy and help-seeking attitudes outcomes were evaluated using the same measure and results were not presented separately, precluding conclusions about whether this improvement was predominantly because of changes in knowledge or attitudes.

The only large randomized controlled trial measured uptake of services and found the use of a website coupled with a telephone coach, reduced uptake of services for the child’s behavior, whilst simultaneously improving child behavior compared with a control group at 24 months follow up [47]. Despite the widespread use of websites and apps to help parents understand their child’s mental health or find services to help their child, only one study evaluated a universally accessible website [43]. Of the 5 studies, 2 (40%) had a comparison group, and neither of these studies compared the DHI to an existing and previously evaluated face-to-face, web-based, or school-based intervention. Thus, the comparative efficacy, feasibility, and cost-effectiveness of DHIs and face-to-face interventions remain unclear.

Of the 5 studies, 2 (40%) reported using theory to inform the design of the DHI. Although there is no evidence to definitively support the use of theory in designing a DHI, it is recommended to use a theory, or theories, to inform the design of health promotion interventions, and it may be beneficial for DHIs targeting help seeking [42,51].

None of the studies reported health economic outcomes of the interventions, such as development costs, implementation expenses, or potential financial benefits from the intervention on the family or health services. The overall quality of the papers was low, with only 20% (1/5) of the studies being a randomized controlled trial. All studies were rated as either high risk of bias on the revised Cochrane tool or serious or critical risk of bias on the Risk of Bias in Nonrandomized Studies of Interventions tool.

In addition, the lack of consistent and validated measures made a meta-analysis impossible and limited our ability to compare efficacy among the interventions. The lack of consistent measures has been described previously [24].

This is the only review showing the impact of DHIs on mental health literacy, help seeking, and referral uptake in parents of children with mental health problems. We searched a wide range of databases, hand searched references from included articles, and attempted to contact authors where data were missing. This study included all quantitative studies evaluating a DHI across multiple time points and thus presented a wider scope of included study designs than existing review articles on DHIs for mental health literacy or help seeking. Finally, this was the only study that extracted data on the theoretical basis of the intervention and economic outcomes.

### Limitations

We included only studies with quantitative outcome measures. We recognize that we could have used categorical coding of qualitative data (eg, positive, neutral, or negative impact) to include qualitative research. This could be an area for future research. In addition, qualitative studies may provide more nuanced data into the effectiveness or otherwise of DHIs in this area, particularly on factors influencing help-seeking attitudes, intentions, and behaviors. In addition, a single reviewer (DP) evaluated all search results against the inclusion and exclusion criteria, which may have resulted in studies being missed at the screening stage. However, hand searching of references within these papers revealed no new studies, suggesting that it is unlikely that we missed any published studies. The studies included were of poor quality; therefore, the results must be interpreted with caution. This review only included peer-reviewed journals and did not include a search for gray literature. As such, there is potential for publication bias in the results. Finally, a lack of consistent nomenclature around help seeking and uptake of services may have resulted in the search strategy missing some studies that measured these outcomes.

### Impact

There is no high-quality evidence that DHIs improve parent mental health literacy, help seeking, or uptake of services, even for the most studied area of ADHD. There is low-quality evidence that parents’ mental health literacy can be improved through the use of DHIs. There is also evidence that the use of a website and telephone coach may reduce the long-term uptake of mental health services for preschool children with disruptive behavior. The economic benefit of any DHI targeting parent mental health literacy, help seeking, or uptake of services remains unknown. This study cautiously supports the use of DHIs, especially ADHD websites, to improve parent mental health literacy. There is no evidence that any DHI can improve help seeking or uptake of services for children with a mental health problem.

### Future Research

Despite the widespread availability, enthusiasm for, and use of DHIs among parents, there is little rigorous evidence regarding the effect of DHIs on parent mental health literacy, help seeking, and uptake of services for their children. There is an urgent need to develop, implement, and rigorously evaluate DHIs designed to improve these outcomes, including an economic evaluation of their effects. Websites targeting parent mental health literacy, especially for mental health problems other than ADHD, should be evaluated to establish whether they increase mental health literacy. Ideally, this evaluation would compare new and previously evaluated interventions using validated measures of parent mental health literacy.

Researchers should conduct randomized controlled trials of new and existing DHIs, including existing interventions that are already frequently accessed by parents. Comparison of face-to-face and school- or community-based interventions would also prove helpful in understanding the role of DHIs within the broader context of child mental health services [25]. Outcomes should include validated measures of parents’ knowledge of mental health problems in children and mental health actions, such as help seeking and uptake of services [25]. Consistent use of validated measures would allow a comparison of interventions and meta-analysis of their effects [52]. Research focusing on help seeking and uptake of services is especially important, given that so many children with mental health disorders are not receiving professional help. Until such research is conducted, we do not know whether a DHI can improve the uptake of mental health services among parents of children with mental health problems. A systematic review of qualitative studies may provide additional information on the influence of DHIs on parents’ help-seeking behaviors.

### Conclusions

This review found low-quality evidence that DHIs may increase mental health literacy for ADHD and increase mental health literacy and help-seeking attitudes toward anxiety and depression. Overall, the heterogeneity of measures and high risk of bias across studies impacted our ability to confidently interpret these findings. We highlight the gap between parents’ frequent use of web-based sources of health information and the paucity of published evidence on the effect of these DHIs on help seeking, the uptake of services, and cost-effectiveness.

## Appendix

Multimedia Appendix 1 Search strategy.

## Abbreviations

ADHD: attention-deficit/hyperactivity disorder
DHI: digital health intervention
PRISMA: Preferred Reporting Items for Systematic Reviews and Meta-Analyses
