# Supplementary material for: The Effect of Digital Health Interventions on Parents’ Mental Health Literacy and Help Seeking for Their Child’s Mental Health Problem: Systematic Review
Source: J Med Internet Res. 2022 Feb 10;24(2):e28771. doi: 10.2196/28771 (PMC8874802; doi:10.2196/28771)
Supplement: Multimedia Appendix 1 [file jmir_v24i2e28771_app1.docx]

**Multimedia Appendix 1. Search Strategy**

**Table S1**. Search concepts and PubMed search terms

| Concept | Description | Search term |
| --- | --- | --- |
| Concept 1 | Parents or caregivers | (Parent* OR caregiver* OR mother* OR father*) |
| Concept 2 | Digital health intervention | (Internet OR software OR mobile app* OR social-media OR social network* OR electronic mail OR email OR e-mail OR mobile phone or smart-phone OR smartphone OR digital OR web-site OR web-based OR world-wide-web OR e-health OR ehealth OR mhealth OR m-health OR online OR e-therapy OR etherapy OR technology*) |
| Concept 3 | Mental health problem | (Mental disorder* OR mental health OR anxiety OR disruptive-behavior OR disruptive-behaviour OR conduct-disorder OR impulse-control OR Mood-disorder* OR depress* OR ADHD OR ADD OR attention-deficit* OR hyperactivity OR child-behavior OR child-behaviour) |
| Concept 4a | Mental health literacy | (Health-education OR health-information OR consumer-health OR health-literacy OR health-promotion OR patient-education OR education OR health-knowledge) |
| Concept 4b | Help-seeking | (Help-seek* OR seeking-behavior OR seeking-behavior OR seeking-treatment OR seek-treatment OR seek-help OR seeking-help OR seeking-intention* OR seeking-attitude*) |
| Concept 4c | Uptake of a mental health service | (Utilisation OR utilization OR usage OR service-use OR treatment-use OR uptake) |

**Concepts 1, 2, AND 3 combined (AND) with each of 4a, 4b, OR 4c.**

(((((Parent* OR caregiver* OR mother* OR father*) AND (Internet OR software OR mobile app* OR social-media OR social network* OR electronic mail OR email OR e-mail OR mobile phone OR smart-phone OR smartphone OR digital OR web-site OR web-based OR world-wide-web OR e-health OR ehealth OR mhealth OR m-health OR online OR e-therapy OR etherapy OR technology*) AND (Mental disorder* OR mental health OR anxiety OR disruptive-behavior OR disruptive-behaviour OR conduct-disorder OR impulse-control OR Mood-disorder* OR depress* OR ADHD OR ADD OR attention-deficit* OR hyperactivity OR child-behavior OR child-behaviour) AND (Health-education OR health-information OR consumer-health OR health-literacy OR health-promotion OR patient-education OR education OR health-knowledge) AND (NOTNLM OR publisher[sb] OR inprocess[sb] OR pubmednotmedline[sb] OR indatareview[sb] OR pubstatusaheadofprint)) AND ("2000/01/01"[PDat] : "3000/12/31"[PDat]) AND English[lang])) OR (((Parent* OR caregiver* OR mother* OR father*) AND (Internet OR software OR mobile app* cR social-media OR social network* OR electronic mail OR email OR e-mail OR mobile phone OR smart-phone OR smartphone OR digital OR web-site OR web-based OR world-wide-web OR e-health OR ehealth OR mhealth OR m-health OR online OR e-therapy OR etherapy OR technology*) AND (Mental disorder* OR mental health OR anxiety OR disruptive-behavior OR disruptive-behaviour OR conduct-disorder OR impulse-control OR Mood-disorder* OR depress* OR ADHD OR ADD OR attention-deficit* OR hyperactivity OR child-behavior OR child-behaviour) AND (Help-seek* OR seeking-behavior OR seeking-behavior OR seeking-treatment OR seek-help OR seeking-help OR seeking-intention* OR seeking-attitude*) AND (NOTNLM OR publisher[sb] OR inprocess[sb] OR pubmednotmedline[sb] OR indatareview[sb] OR pubstatusaheadofprint)) AND ("2000/01/01"[PDat] : "3000/12/31"[PDat]) AND English[lang])) OR (((Parent* OR caregiver* OR mother* OR father*) AND (Internet OR software OR mobile app* OR social-media OR social network* OR electronic mail OR email OR e-mail OR mobile phone OR smart-phone OR smartphone OR digital OR web-site OR web-based OR world-wide-web OR e-health OR ehealth OR mhealth OR m-health OR online OR e-therapy OR etherapy OR technology*) AND (Mental disorder* OR mental health OR anxiety OR disruptive-behavior OR disruptive-behaviour OR conduct-disorder OR impulse-control OR Mood-disorder* OR depress* OR ADHD OR ADD OR attention-deficit* OR hyperactivity OR child-behavior OR child-behaviour) AND (Utilisation OR utilization OR usage OR service-use OR treatment-use OR uptake) AND (NOTNLM OR publisher[sb] OR inprocess[sb] OR pubmednotmedline[sb] OR indatareview[sb] OR pubstatusaheadofprint)) AND ("2000/01/01"[PDat] : "3000/12/31"[PDat]) AND English[lang])

**CINAHL search terms**

(((MH "Parents+") or (MH "Caregivers") or TX mother* or father*) and ((MH "Internet+") or (MH "Software") or (MH "Mobile Applications") or (MH "Social Media") or
(MH "Cellular Phone+") or TI (digital or web* or internet or e-health or ehealth or mhealth or m-health or online or etherapy or e-therapy) OR AB (digital or web* or internet or e-health or ehealth or mhealth or m-health or online or etherapy or e-therapy) ) And (MH "Health Education") or (MH "Health Information+") or (MH "Health Promotion") or (MH "Patient Education") OR (MH "Health Education") OR (MH "Parenting Education") or TX patient-acceptance or
(MH "Health Services Accessibility") or (MH "Help Seeking Behavior") OR (MH "Information Seeking Behavior") or TX (health* N2 (behavio?r* or access*) or mental-health-literacy or (mental-health N2 awareness) or (seek* N1 treatment*) or (help N2 seek*) or (service N2 (utili?ation or usage or "use" or uptake)) or TI education OR AB education or (MH "Health Knowledge")) And ((MH "Mental Disorders, Chronic") OR (MH "Mental Disorders Diagnosed in Childhood") OR (MH "Mental Disorders") OR (MH "Attention Deficit Hyperactivity Disorder") OR (MH "Child Behavior Disorders") OR (MH "Separation Anxiety") OR (MH "Social Anxiety Disorders") or (MH "Anxiety") OR (MH "Dental Anxiety") OR (MH "Separation Anxiety") or (MH "Anxiety Disorders") OR (MH "Depression") OR (MH "Generalized Anxiety Disorder") OR (MH "Panic Disorder") OR (MH "Phobic Disorders+") OR (MH "Social Anxiety Disorders") or (MH "Disruptive Behavior") OR (MH "Child Behavior") or (MH "Neurotic Disorders") or (MH "Affective Disorders") or (MH "Affective Symptoms") or TX conduct-disorder* or TX oppositional-defiant) Expanders Apply Equivalent subjects, Search modes: Boolean/Phrase) OR (((MH "Internet+") or (MH "Software") or (MH "Mobile Applications") or (MH "Social Media") or (MH "Cellular Phone+") or TI (digital or web* or internet or e-health or ehealth or mhealth or m-health or online or etherapy or e-therapy) OR AB (digital or web* or internet or e-health or ehealth or mhealth or m-health or online or etherapy or e-therapy)) and ((MH "Health Education") or (MH "Health Information+") or (MH "Health Promotion") or (MH "Patient Education") OR (MH "Health Education") OR (MH "Parenting Education") or TX patient-acceptance or
(MH "Health Services Accessibility") or (MH "Help Seeking Behavior") OR (MH "Information Seeking Behavior") or TX (health* N2 (behavio?r* or access*) or mental-health-literacy or (mental-health N2 awareness) or (seek* N1 treatment*) or (help N2 seek*) or (service N2 (utili?ation or usage or "use" or uptake)) or TI education OR AB education or (MH "Health Knowledge")) and ((MH "Mental Disorders, Chronic") OR (MH "Mental Disorders Diagnosed in Childhood") OR (MH "Mental Disorders") OR (MH "Attention Deficit Hyperactivity Disorder") OR (MH "Child Behavior Disorders") OR (MH "Separation Anxiety") OR (MH "Social Anxiety Disorders") or (MH "Anxiety") OR (MH "Dental Anxiety") OR (MH "Separation Anxiety") or (MH "Anxiety Disorders") OR (MH "Depression") OR (MH "Generalized Anxiety Disorder") OR (MH "Panic Disorder") OR (MH "Phobic Disorders+") OR (MH "Social Anxiety Disorders") or (MH "Disruptive Behavior") OR (MH "Child Behavior") or (MH "Neurotic Disorders") or (MH "Affective Disorders") or (MH "Affective Symptoms") or TX conduct-disorder* or TX oppositional-defiant) Limiters: Age groups: All adult, Expanders Apply Equivalent subjects, Search modes: Boolean/Phrase) Limiters: Peer reviewed)

**Embased search terms**

1. exp parent/ or caregiver/

2. (mother*1 or father*1).tw,kw,dq.

3. internet/ or software/ or exp mobile application/ or social media/ or exp social network/

4. e-mail/ or exp mobile phone/

5. (digital or web or internet or ehealth or e-health or mhealth or m-health or online or e-therapy).tw,kw,dq.

6. patient-acceptance.tw,kw,dq.

7. health education/ or health literacy/ or exp health promotion/ or parenting education/ or patient education/ or psychoeducation/ or school health education/ or consumer health information/ or health care delivery/ or help seeking behavior/

8. ((health* adj3 (behavio?r* or access*)) or mental-health-literacy or (mental-health adj3 awareness) or (seek* adj2 treatment*) or (help adj3 seek*) or (service adj3 (utili#ation or usage or "use" or uptake))).tw,kw,dq.

9. mental disease/ or exp anxiety disorder/ or exp behavior disorder/ or exp emotional disorder/ or exp mood disorder/

10. conduct disorder/

11. exp child behavior/

12. education.tw,kw,dq,hw.

13. attitude to health/

14. awareness/

15. (1 or 2) and (3 or 4 or 5) and (9 or 10 or 11) and (6 or 7 or 8 or 12 or 13 or 14)

16. (*internet/ or *software/ or exp *mobile application/ or (*e-mail/ or exp *mobile phone/) or 5) and (*mental disease/ or exp *anxiety disorder/ or exp *behavior disorder/ or exp *emotional disorder/ or exp *mood disorder/ or *conduct disorder/ or exp *child behavior/) and (6 or (*health education/ or *health literacy/ or exp *health promotion/ or *parenting education/ or *patient education/ or *psychoeducation/ or *school health education/ or *consumer health information/ or *health care delivery/ or *help seeking behavior/) or 8 or 12 or *attitude to health/ or *awareness/)

17. limit 16 to adult <18 to 64 years>

18. 15 or 17

19. limit 18 to (conference abstract or conference paper or "conference review" or editorial or letter)

20. 18 not 19

**Medline search terms**

1. exp Parents/ or caregivers/

2. (mother*1 or father*1).tw,kf.

3. exp internet/ or software/ or mobile applications/ or social media/ or exp social networking/

4. electronic mail/ or exp cell phone/

5. (digital or web* or internet or e-health or ehealth or mhealth or m-health or online or etherapy or e-therapy).tw,kf.

6. health education/ or consumer health information/ or health literacy/ or health promotion/ or patient education as topic/ or "patient acceptance of health care"/ or health services accessibility/

7. help-seeking behavior/

8. ((health* adj3 (behavio?r* or access*)) or mental-health-literacy or (mental-health adj3 awareness) or (seek* adj2 treatment*) or (help adj3 seek*) or (service adj3 (utili#ation or usage or "use" or uptake))).tw,kf.

9. mental disorders/ or exp anxiety disorders/ or exp "disruptive, impulse control, and conduct disorders"/ or exp mood disorders/ or anxiety/ or dental anxiety/ or depression/ or mental health/

10. "attention deficit and disruptive behavior disorders"/ or attention deficit disorder with hyperactivity/ or conduct disorder/ or child behavior disorders/

11. education.tw,kf,hw.

12. health knowledge, attitudes, practice/ or awareness/

13. (1 or 2) and (3 or 4 or 5) and (6 or 7 or 8 or 11 or 12) and (9 or 10)

14. (exp *internet/ or *software/ or *mobile applications/ or (*electronic mail/ or exp *cell phone/) or 5) and (*health education/ or *consumer health information/ or *health literacy/ or *health promotion/ or *patient education as topic/ or *"patient acceptance of health care"/ or *health services accessibility/ or *help-seeking behavior/ or 8 or 11 or (*health knowledge, attitudes, practice/ or *awareness/)) and (*mental disorders/ or exp *anxiety disorders/ or exp *"disruptive, impulse control, and conduct disorders"/ or exp *mood disorders/ or *anxiety/ or *dental anxiety/ or *depression/ or *mental health/ or (*"attention deficit and disruptive behavior disorders"/ or *attention deficit disorder with hyperactivity/ or *conduct disorder/ or *child behavior disorders/))

15. limit 14 to "all adult (19 plus years)"

16. 13 or 15

17. limit 16 to (case reports or editorial or letter)

18. 16 not 17

**PsycINFO search terms**

1. exp parents/ or caregivers/ or (mother*1 or father*1).ti,ab,id.

2. exp internet/ or electronic health services/ or digital interventions/ or mobile health/ or exp social media/ or online social networks/

3. (digital or web* or internet or ehealth or e-health or mhealth or m-health or online or etherapy or e-therapy).ti,ab,id.

4. exp health literacy/ or mental health literacy/ or health knowledge/ or health information/ or health promotion/ or client education/

5. exp "information and communication technology"/

6. health care delivery/ or mental health services/ or health care services/ or health care utilization/

7. exp help seeking behavior/

8. ((health* adj3 (behavio?r* or access*)) or mental-health-literacy or (mental-health adj3 awareness) or (seek* adj2 treatment*) or (help adj3 seek*) or (service adj3 (utili#ation or usage or "use" or uptake))).ti,ab,id.

9. attention deficit disorder/ or attention deficit disorder with hyperactivity/ or behavior problems/ or tantrums/ or child behavior/

10. exp behavior disorders/ or exp affective disorders/ or exp anxiety disorders/ or exp anxiety/ or exp stress/

11. education.ti,ab,id,hw. or health knowledge/

12. exp "Depression (Emotion)"/

13. 1 and (2 or 3 or 5) and (9 or 10 or 12) and (4 or 6 or 7 or 8 or 11)

14. (2 or 3 or 5) and (9 or 10 or 12) and (4 or 6 or 7 or 8 or 11)

15. limit 14 to "300 adulthood "

16. 13 or 15

17. limit 16 to (editorial or letter or obituary or poetry or review-book)

18. 16 not 17
